# Evaluating the quality of online patient education materials for gastric adenocarcinoma

**DOI:** 10.3389/fdgth.2026.1699285

**Published:** 2026-04-01

**Authors:** Trisha Ray, Taylor Barrow, Lauren Hamel, Najeeb Al Hallak, Asfar S. Azmi, Anthony Shields, Steve Kim, Miguel Tobon, Eliza W. Beal

**Affiliations:** 1 Wayne State University School of Medicine, Detroit, MI, United States; 2 Department of Oncology, Karmanos Cancer Institute/Wayne State University School of Medicine, Detroit, MI, United States; 3 Department of Surgery, Karmanos Cancer Institute/Wayne State University School of Medicine, Detroit, MI, United States

**Keywords:** cancer, gastric adenocarcinoma, gastroenterology, oncology, online patient education materials, public health, screening, stomach cancer

## Abstract

**Background:**

Gastric adenocarcinoma, or gastric cancer, typically has a poor prognosis. The objective of this study was to assess the quality, understandability, actionability, and comprehensiveness of online resources for patients diagnosed with gastric adenocarcinoma, or gastric cancer as patients increasingly rely on online health information.

**Methods:**

A systematic search using the term “stomach cancer” was conducted across three search engines (Google, Yahoo, and Bing) on three different browsers (Safari, Google Chrome, and Microsoft Edge) on 12/13/2024, with the top fifty websites recorded for each combination. Duplicates were removed and inclusion/exclusion criteria were applied. Quality was evaluated using the DISCERN instrument. The PEMAT-P was used to evaluate understandability and actionability. Readability was evaluated with the Flesch-Kincaid Reading Ease algorithm. Comprehensiveness was evaluated with author generated criteria based on national guidelines. Scores for each assessed metric were determined by two independent reviewers for each website and recorded, with any inter-reviewer discrepancies resolved by consensus. Statistical analysis was performed to compare results by website affiliation (academic, foundation or government) and search rank.

**Results:**

Thirty-seven websites evaluated (*N* = 17 academic, *N* = 13foundation and *N* = 7 government). The mean quality score (DISCERN) was 3.62 (SD 1.21), with no significant differences across affiliations or search positions. Thirty-five out of the 37 evaluated websites achieved an understandability (PEMAT-P) score above the recommended threshold of 70% (Mean 78.38%, SD 11.86%) and 14 websites exceeded the threshold for actionability (Mean 57.66%, SD 37.69%) with no significant differences across affiliations or search positions. Readability (Flesch-Kincaid) averaged a 10th–12th grade level, with a mean score of 51.88 (SD 8.93). Mean comprehensiveness was scored at 62.98% (SD 23.23%) across all websites without significant differences across affiliations or search positions, with over 85% of websites addressing epidemiology, risk factors, and symptomatology, but under 30% of websites including content on post-treatment complications or surveillance.

**Conclusions:**

While most online resources for gastric cancer provided understandable information, they lacked actionability, were written above recommended reading levels, and offered limited content on long-term management. These shortcomings reflect broader trends seen across other patient resources and highlight the need for more actionable, readable, and comprehensive online patient education materials.

## 1 Introduction

Gastric adenocarcinoma, or gastric cancer, is the most common histologic type of stomach cancer, arising from the glandular epithelium of the gastric mucosa (1). Worldwide, gastric cancer accounts for 7% of cancer diagnoses, making it the 5th most common cancer globally, accounting for 9% of cancer-related deaths (2). Although gastric cancer is relatively uncommon in the United States, comprising about 1.5% of new cancer diagnoses annually, the prognosis for gastric cancer remains poor as most cases are diagnosed at an advanced stage (1, 2). The five-year survival rate in the United States is around 20%, and for advanced or metastatic disease, median survival is less than one year (3, 4). Gastric cancer presents with variable symptoms including dyspepsia, weight loss, and loss of appetite, or may be asymptomatic (5). Treatment of gastric cancer is multimodal and often includes systemic chemotherapy and surgery for early stage disease and may include chemotherapy and immunotherapy for later stage disease (6).

Online resources have become a primary source of healthcare information for patients. Recent studies indicate that 58.5% of patients seek medical information online (7–9). In a survey investigating the patterns of users seeking health information online, the most common first source used to answer health questions was search engines, with 95% of participants reporting use of search engines such as Google, Bing, and Yahoo (7–11). Among adults ages 18–44, the use of the Internet to search for health or medical information exceeds 60% (8). Across various demographic groups, individuals most frequently search for symptoms, diagnoses, and treatment options (9). In addition, generative artificial intelligence (AI) tools are increasingly shaping how patients access health information. These tools rely on websites as the primary sources from which they retrieve and synthesize health-related content, pulling information from various online resources, such as medical websites and articles, and generating answers based on the data they access (12). As a result, the reliability of the AI-generated responses depends directly on the quality of the sources used, further underscoring the importance of evaluating the original online content and the need for careful consideration of how these tools influence patient understanding and decision-making. Further, the quality and readability of online health information are highly variable, as some online resources may be difficult for the average patient to understand, may display only a limited scope of information, or contain inaccurate or incomplete information, which can limit their educational value and potentially affect patients' understanding of their diagnosis. To ensure these resources are accessible for the general public, it is recommended by the National Institutes of Health, the US Department of Health and Human Services, and the American Medical Association that patient education materials be written at a sixth-grade reading level, as this improves comprehension for a broader audience (13). The knowledge gained by patients through online research can significantly enhance their understanding of their condition, enabling them to be more engaged in discussions with their healthcare providers and more proactive in managing their treatment plans, which can lead to improved treatment adherence and outcomes (14). Therefore, assessing the quality, understandability, actionability, readability, and comprehensiveness of online patient education materials is essential to ensure that patients are provided with reliable medical information and are empowered to make informed decisions about their diagnoses and care.

The domains of website quality, understandability, actionability, readability, and comprehensiveness were selected for investigation in this study because they represent fundamental elements for assessing high-quality health information. Several existing, validated tools can be used to aid in the evaluation of online health information. Quality in consumer health information is a multidimensional concept that goes beyond readability and presentation, encompassing accuracy, completeness, and the ability to support informed decision-making through reliable, evidence-based content (15). A systematic review of online health information quality found that trustworthiness, expertise, and objectivity constitute core dimensions of how consumers judge the quality of health websites (16). The DISCERN tool is well suited to assess this concept because it provides a standardized, validated framework for evaluating written information by focusing on reliability, clarity, and the presence of evidence-based guidance. Assessing quality is crucial for patient education resources because having access to accurate and trustworthy information is important for patients to be able to make informed treatment decisions, and without such standards, the rapid growth of online medical information makes it difficult to identify which resources are safe and useful (15).

Understandability refers to the extent to which users can grasp and explain the key messages of patient education materials, while actionability refers to the extent to which users can identify clear, practical steps to act on the information provided (17). A systematic review of online cardiovascular disease risk calculators identified understandability and actionability as core metrics to evaluate patient resources because most calculators scored low on these domains, indicating users may struggle both to grasp key messages and to identify what actions to take (18). The Patient Education Materials Assessment Tool (PEMAT) is a validated instrument that assesses these aspects and enables evaluation of how well materials support comprehension and real-world application (17). This is essential for evaluating patient education resources because effective health communication depends not only on clarity but also on whether users can translate information into tangible next steps, making understandability and actionability central to high-quality, impactful health information (17).

Readability refers to the ease with which a reader can understand written material, determined by the level of reading comprehension required to process the information (19). In a 2018 systematic review, readability was identified as a central quality metric of online health information, as it was demonstrated that content written above the public's reading ability undermines its accessibility and effectiveness (20). The Flesch-Kincaid model is a validated and widely used tool that measures readability by estimating the U.S. academic grade level needed to comprehend a text, making it a reliable method for assessing the complexity of patient education materials. Assessing readability is essential for evaluating patient education resources because patients' ability to use online health information to make informed decisions depends on comprehension. Materials written above recommended reading levels may be inaccessible to many users, and lower health literacy has been found to be linked to higher rates of hospitalization, poorer health outcomes, and increased costs (19).

Comprehensiveness refers to the inclusion of all relevant information related to a health condition, covering aspects such as incidence, epidemiology, risk factors, presentation, genetic predisposition and screening, workup, staging, surgery, systemic therapy, post-treatment complications, prognosis and survival, and surveillance. A systematic review of instruments used to assess online health information found comprehensiveness to be a core criterion, because incomplete information can leave patients inadequately informed for decision-making (21). Using current national guidelines to assess comprehensiveness is an effective approach, as these guidelines are evidence-based, up-to-date, and reflect the most accurate standards of care established by relevant medical societies. Evaluating patient education resources for comprehensiveness is crucial, as it ensures patients receive the full scope of necessary information, enabling them to understand their condition, make informed decisions, and effectively manage their health in collaboration with their healthcare providers (22).

Prior studies assessing the accuracy and readability of online health information for patients diagnosed with pancreatic cancer have noted that many web-based resources exceed recommended readability standards, often requiring collegiate-level comprehension, and are therefore inaccessible to a large portion of the general population (23). While higher readability levels were frequently associated with greater informational accuracy, accuracy was found to vary by content type (23). Websites describing standard treatment modalities were generally reliable, whereas those focused on alternative therapies showed a higher prevalence of inaccuracies, reflecting both differences in site affiliation and the underrepresentation of alternative therapies on academic and government platforms (23). In the assessment of cancer-related patient education materials from Canadian provincial cancer agencies, encompassing various cancer types such as breast cancer and colon cancer, quality, evaluated using DISCERN, revealed notable deficits in accountability and decision-support content, including poor reporting of sources and currency, limited discussion of treatment alternatives, and minimal explanation of outcomes without treatment (24). Readability analyses showed that all provinces exceeded the recommended 7th-grade reading level, with only two provinces below the national 9th-grade average, and there was frequent use of complex, commonly misunderstood medical terms without adequate definitions (24). Actionability assessed by PEMAT was also limited, with only two provinces meeting the ≥70% threshold, largely due to the lack of visual aids, tangible action tools, and summary sections to support patient understanding and next steps (24). In a study evaluating readability and quality of colorectal cancer online patient education resources, it was found that the websites demonstrated moderate readability, with a mean Flesch-Kincaid grade level of 6.9 (25). However, the mean DISCERN score of 52.2 indicated only moderate and potentially unreliable quality, with government-certified websites performing better than non-certified sites (25).

**Figure 1:**
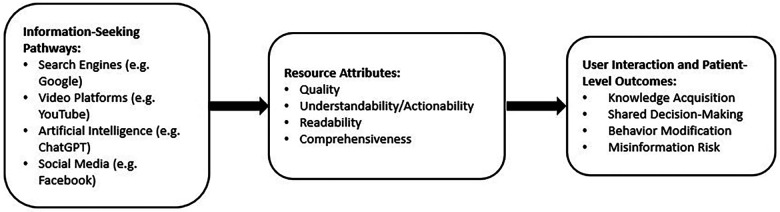
Conceptual framework for gastric adenocarcinoma patient education resources, linking information-seeking pathways, resource attributes, and user interaction.

While these prior works have assessed metrics such as readability, quality, actionability, understandability, and comprehensiveness of online patient education materials for cancers such as breast and pancreatic cancer, a more recent large-scale, comprehensive evaluation of these domains specifically within gastric cancer resources is lacking. One existing study evaluated online materials for esophageal, gastric, and colorectal cancer treatment by comparing English and Spanish content using PEMAT to evaluate understandability and actionability and DISCERN to evaluate quality, and found consistently high reading grade levels and variable actionability (26). However, it did not isolate gastric cancer content for an in-depth analysis and omitted key measures such as readability and comprehensiveness, leaving a critical gap in understanding the unique informational needs and deficiencies faced by gastric cancer patients (26). Further, in a 2011 study, it was found that gastric cancer websites generally had poor readability, with an average reading level around 10th grade, which is higher than recommended for patient materials. This study also found that completeness and accuracy of information were only “moderate” overall, but did not evaluate understandability and actionability, nor comprehensively assess website quality. Additionally, this study was conducted over ten years ago and may not reflect the current web environment and updated gastric cancer guidelines (27). An additional recent study assessed the quality and readability of online gastric cancer information using validated tools and found widespread deficiencies, including poor integrity, incomplete discussion of treatment risks and quality-of-life impacts, low overall quality scores, and readability levels that exceeded recommended standards for patient education (28). However, because the analysis was limited to a small number of websites from select search engines and did not evaluate metrics such as understandability or actionability, there remains a clear need for broader evaluations of gastric cancer education resources across multiple metrics (28). This study aims to evaluate the quality, understandability, actionability, readability, and comprehensiveness of online patient education materials for individuals diagnosed with gastric cancer, to identify key gaps that may be utilized to improve on their effectiveness in supporting patient understanding and decision-making (Figure 1).

## 2 Materials and methods

### 2.1 Search method

In this cross-sectional study, searches were conducted by a single user across three search engines (Google, Yahoo, and Bing) on three different browsers (Safari, Google Chrome, and Microsoft Edge) on November 12, 2024. These tools were chosen as they are some of the most popular platforms for online information gathering (11, 29, 30). Private browsing mode was used in each search engine to minimize targeted search responses and website data tracking, including physical location and search history. To streamline the search technique, the search term “stomach cancer” was utilized across all platforms. Data organization and compilation were performed using Google Sheets with archival of all website content as it was presented on this date.

### 2.2 Website selection

**Figure 2:**
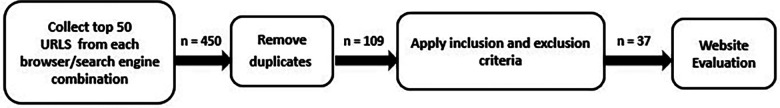
Website selection process for gastric adenocarcinoma patient information.

The URLs of the top 50 websites identified by each browser and search engine combination were recorded in a spreadsheet, as this number of search results best reflects patient search behavior and provides a practical balance between representativeness and feasibility. The positional rank and number of appearances across search engines and browsers were recorded for each website. Duplicates across platforms were then removed. A list of inclusion and exclusion criteria was applied to each website, and websites not meeting these criteria were removed (Table 1). Websites were then further categorized by affiliation and position and evaluated on quality, readability, understandability, actionability, and comprehensiveness, from March 5th, 2025 – April 16th, 2025 (Figure 2).

**Table 1:** Inclusion and exclusion criteria.

Inclusion criteria	Exclusion criteria
Provides information specific to diagnosis and management of gastric adenocarcinoma (gastric cancer) Targeted towards patients. Accessible to the general population i.e., no subscription/fee needed. English Language	Website does not provide information specific to gastric adenocarcinoma. Pharmaceutical advertisement Fundraiser Blogs, mailing lists, social media, discussion boards. Advertisement without any information Fails to link to objective resources with empirical evidence. Too generalized Website inaccessibility (error or under construction) Audiovisual content (videos, podcasts) without accompanying text otherwise meeting inclusion criteria. No unique information – links to published articles without providing information, links to other websites without providing information. Links to research articles not published in academic journals

### 2.3 Website evaluation

#### 2.3.1 Affiliation

Website affiliations were assigned based on the following criteria:

- Academic: The website must be published by an accredited university, post-secondary school, or academic healthcare center/hospital. Additionally, peer-reviewed journal articles that a government-funded website has not published will be included in this category.
- Foundation: The website must be published by a registered, medically related organization with 501(c)(3) or equivalent non-profit status.
- Government: The website must be government-funded and medically focused.

#### 2.3.2 Position

Search position was defined as where each web page appeared in the order of search results from top to bottom. The search position was calculated for each browser/search engine combination, and then a mean search position across all combinations was calculated for web pages that appeared in multiple browser/search engine combinations. If two websites had the same mean search position, their respective placement in the position ranking was determined by the number of appearances across the different web browsers and search engines used. After mean search positions were calculated, web pages were split into three groups. The top group contained the first 12 web pages by ranked search position, the second group contained the next 13 web pages, and the last group contained the bottom 12 web pages.

#### 2.3.3 Evaluation of website quality, understandability, actionability, readability and comprehensiveness

Website quality was evaluated using the DISCERN instrument (15). The DISCERN instrument a tool that assesses the quality of written health information, with a focus on patient information on treatment choices (15). It was originally designed for use by patients and providers, with applicability to healthcare professionals and researchers, and was tested and validated through expert panels, information providers, and self-help group members using written materials across multiple medical conditions (15). Its primary purpose is to provide a systematic method for evaluating the reliability and quality of health-related literature, ensuring that patients receive clear, accurate, and unbiased information (15). The DISCERN instrument consists of 15 questions, each rated on a discrete scale of 1–5. The 16th question represents the overall score for the article and is also rated 1–5 based on the ratings for the other questions. Two independent reviewers applied the DISCERN instrument to each article, with each reviewer recording their scores on a shared spreadsheet, and the results were compared. Any inter-rater discrepancies were resolved by discussion and consensus.

The Patient Education Materials Assessment Tool (PEMAT), originally designed for use by healthcare professionals and reviewers to systematically evaluate patient education materials, measures the understandability and actionability of patient education resources. This tool was first developed and validated in 2013 among English-language patient education materials, and subsequently validated across multiple linguistic and cultural contexts, demonstrating its applicability in diverse patient populations and clinical settings (17). Its primary purpose is to evaluate how effectively these materials communicate health information to patients, ensuring that they can comprehend and apply the information meaningfully (31). Each factor is assessed on a binary scale, receiving a score of 0 if absent and 1 if present. The cumulative score is then expressed as a percentage of the maximum possible four points. The understandability aspect assesses whether the material is easy to read and comprehend. It considers factors such as use of plain language, clarity of concepts, organization, flow of information, and use of visuals and formatting (31). The actionability component evaluates whether the material provides clear, specific steps that patients can take. It assesses clarity in instructions, feasibility of the actions described, and encouragement of patient engagement and empowerment (31). Each factor is assessed on a binary scale, receiving a score of 0 if absent and 1 if present. The cumulative score is then expressed as a percentage of the maximum possible three points. Two independent reviewers of each article rated the material against the standardized set of criteria and questions included in the PEMAT, with each reviewer recording their scores on a shared spreadsheet, after which results were compared, and any inter-rater discrepancies were resolved by discussion and consensus.

Readability was assessed using the Flesch-Kincaid model, an assessment tool originally developed in the 1970s for the United States Armed Forces. The Flesch-Kincaid grade level had been previously validated during its original development within U.S. military populations and has since been widely applied and supported in healthcare research contexts, including the assessment of patient education handouts, medical websites, consent forms, and clinical questionnaires (32). This model denotes the grade level the reader would have to be to read a segment of text successfully, based on sentence length and word syllable count (33). The Flesch-Kincaid reading ease (FKRE) algorithm assigns scores from 0 to 100, with 0 being the hardest to read and 100 being the easiest (33). Each score range correlates to a specific reading grade level (i.e., 80.0–90.0 = 6th grade level), with a score above 80.0 correlating to a reading level of 6th grade or below, which aligns with the NIH-recommended threshold (Table 2) (33, 34). Grade levels span from 5th grade to professional (university graduate with expertise in the field). The Flesch-Kincaid grade level for each web page was calculated in Microsoft Word.

**Table 2:** Flesch-Kincaid scores with corresponding reading grade levels.

Score	Estimated reading grade level
90–100	5th grade
80–90	6th grade
70–80	7th grade
60–70	8th–9th grade
50–60	10th–12th grade
30–50	College
0–30	College graduate

Comprehensiveness scores were calculated using a 10-point checklist adapted from National Comprehensive Cancer Network (NCCN) guidelines (Table 3) (35). Categories were scored based on each checklist item mentioned, with any single item mentioned within a category earning the whole category 1 point with a maximum score of 10. This assessment was conducted by two independent reviewers, with each reviewer recording their scores on a shared spreadsheet, following which results were compared, and any inter-rater discrepancies were resolved by discussion and consensus.

**Table 3:** Comprehensiveness criteria for gastric adenocarcinoma.

Category	Sub-topic criteria	
Incidence, epidemiology, risk factors (Yes = 1 point, No = 0 points)	Gender Ethnic background (East Asian, Latin American) Anatomical subsite of disease	H pylori Obesity GERD EBV
Genetic predisposition and screening (Yes = 1 point, No = 0 points)	APC gene FAP gene TP53 gene	Li-Fraumeni syndrome Peutz Jager syndrome
Presentation (Yes = 1 point, No = 0 points)	Asymptomatic Dysphagia Asthenia Indigestion	Vomiting Weight loss Early satiety Iron deficiency anemia
Work-up (Yes = 1 point, No = 0 points)	EGD Biopsy Imaging (CT C/A/P, PET-CT)	EUS if early-stage Biopsy of metastatic disease, if present
Staging (Yes = 1 point, No = 0 points)	AJCC TNM staging	
Surgery (Yes = 1 point, No = 0 points)	Partial gastrectomy	Total gastrectomy
Systemic therapy (Yes = 1 point, No = 0 points)	Perioperative/adjuvant chemotherapy	Immunotherapy Targeted therapy
Post-treatment complications (Yes = 1 point, No = 0 points)	S/p partial or total gastrectomy	S/p FLOT or other chemotherapy
Prognosis and survival (Yes = 1 point, No = 0 points)	Prognosis	Survival
Surveillance (Yes = 1 point, No = 0 points)	CT of thorax/abdomen every 6–12 weeks	
Total possible points = 10		

### 2.4 Statistical analysis

For each of the 5 evaluation criteria (quality, understandability, actionability, readability, and comprehensiveness), variables were approximated as continuous and mean scores and standard deviations were calculated overall and for the foundation, academic, and commercial categories, respectively. A one-way ANOVA test was then conducted using GraphPad (Dotmatics, Boston, MA, USA) to determine significant differences between categories using a standard predetermined *p*-value of 0.05. This same process was then repeated for the three ranked search position categories. For the quality assessment, mean DISCERN scores were calculated across all websites for each question, and the two highest and two lowest scoring questions were recorded. For the comprehensiveness evaluation, mean scores were calculated across all websites for each category, and the three highest and two lowest scoring categories were recorded.

## 3 Results

### 3.1 Website selection

A total of 37 suitable websites were found to meet the pre-defined inclusion and exclusion criteria.

### 3.2 Website affiliation

Upon applying categorization criteria to each of the 37 web pages in the study, there were a total of 17 academic pages, 13 foundation pages, and 7 government pages.

### 3.3 Quality

**Figure 3:**
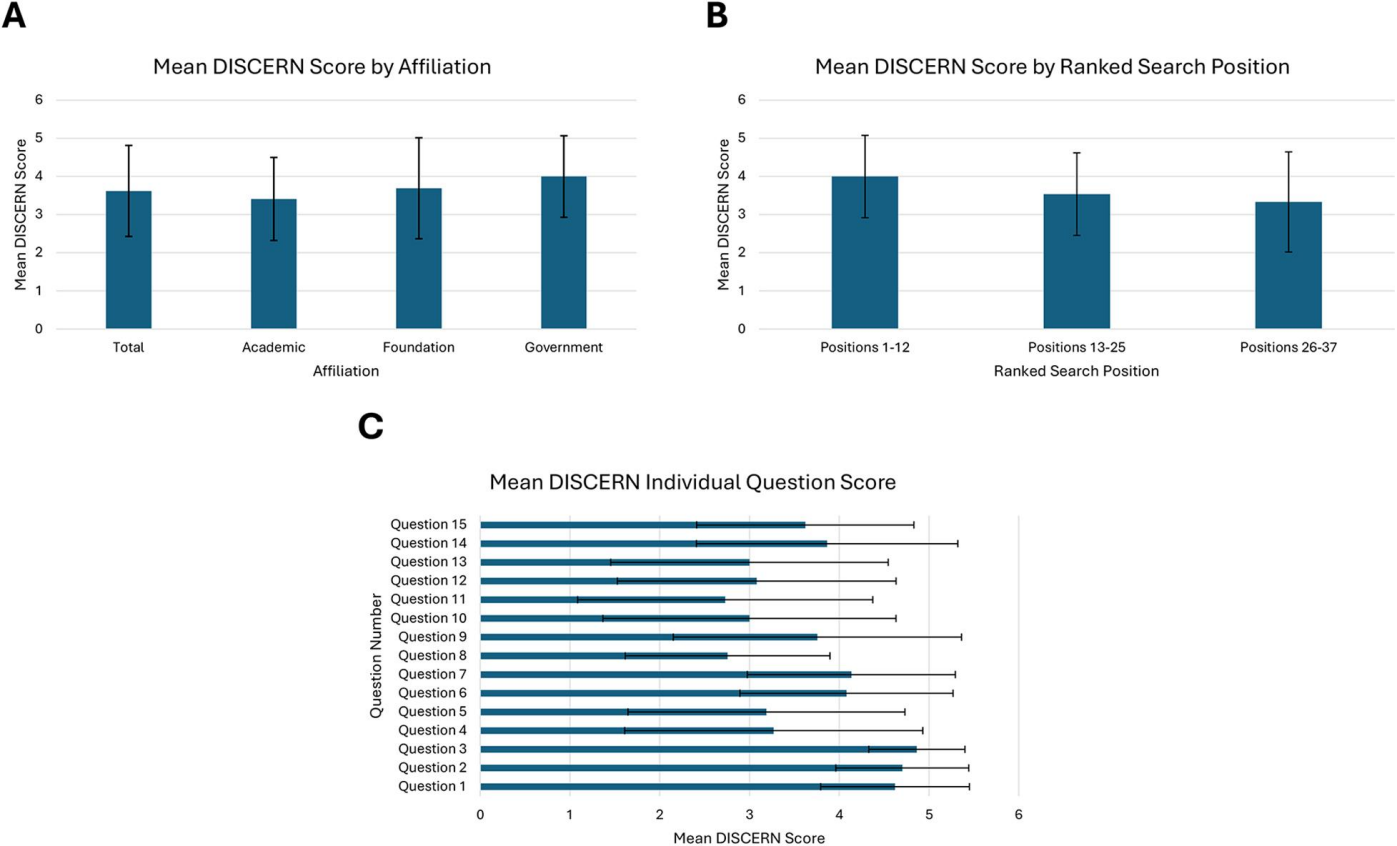
**(A)** Mean total DISCERN score and scores by affiliation (*p* = 0.55), **(B)** mean DISCERN scores by ranked search position (*p* = 0.39), **(C)** mean DISCERN scores for individual questions 1–15. DISCERN scores are expressed on a scale of 1–5.

The mean DISCERN score was 3.62 (SD 1.21) across all evaluated websites. The mean scores for academic, foundation, and government web pages were 3.41 (SD 1.12), 3.69 (SD 1.38), and 4 (SD 1.15), respectively (Figure 3A). No significant differences were present between groups (*p* = 0.55). Between ranked search position groups, the webpages with the top 1–12 average positions had a mean DISCERN score of 4 (SD1.13), the webpages with the top 13–25 positions had a mean score of 3.54 (SD 1.13), and the web pages with the top 26–37 positions had a mean score of 3.33 (SD 1.37). No significant differences were present between groups (*p* = 0.39) (Figure 3B). Across all websites, the two questions with the highest mean DISCERN score were question 2, “Does it achieve its aims?”, with a mean of 4.70 (SD 0.74), and question 3, “Is it relevant?” with a mean score of 4.86 (SD 0.54). The two questions with the lowest mean scores were question 8, “Does it refer to areas of uncertainty?”, with a mean score of 2.76 (SD 1.14), and question 11, “Does it describe what would happen if no treatment is used?”, with a mean score of 2.73 (SD 1.64) (Figure 3C).

### 3.4 Understandability

**Figure 4:**
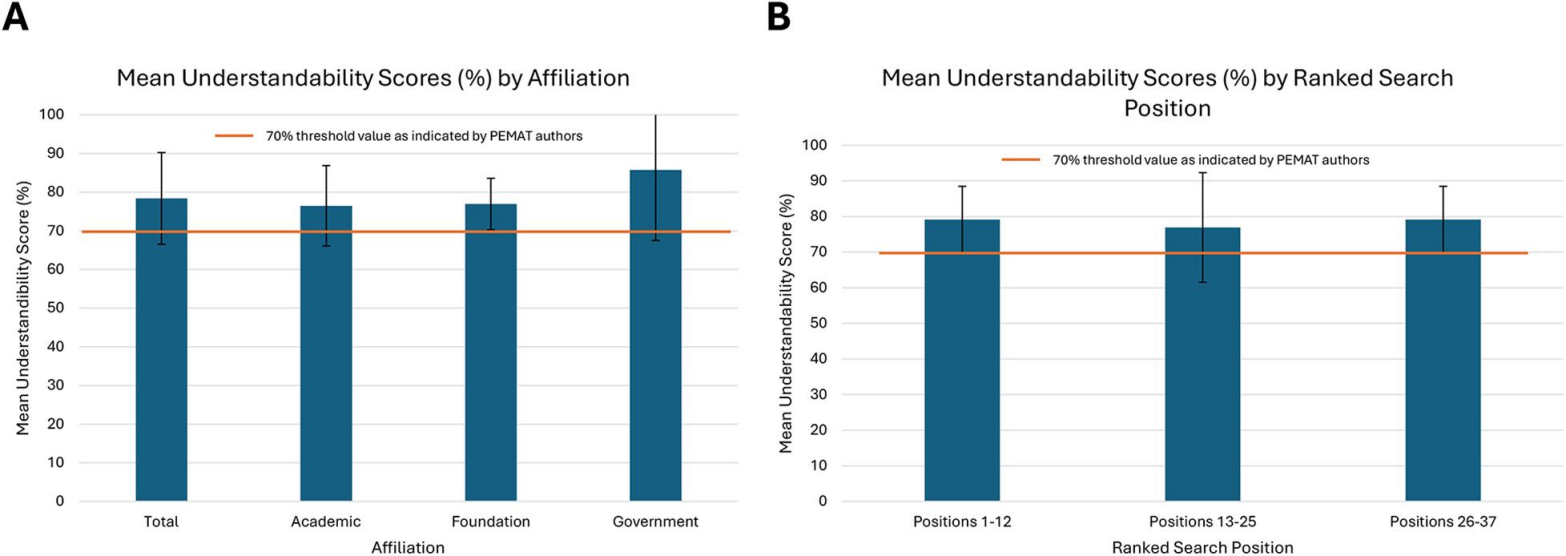
Mean PEMAT understandability scores with 70% recommendation threshold line indicated. **(A)** Mean total Understandability score and scores by affiliation (*p* = 0.20), **(B)** Mean Understandability scores by ranked search position (*p* = 0.87). PEMAT Understandability scores are expressed as a percentage of total criteria met (1–4).

35 out of the 37 websites evaluated exceeded the 70% threshold for an acceptable understandability score using the PEMAT tool. The mean understandability score across all sites was 78.38% (SD 11.86%). The average scores for academic, foundation, and government sites were 76.47% (SD 10.40%), 76.92% (SD 6.66%), and 85.71% (SD 18.21%), respectively (Figure 4A). There were no statistically significant differences between groups (*p* = 0.20). Mean understandability scores for the ranked search position groups were 79.17% (SD 9.32%) for positions 1–12, 76.92% (SD 15.38%) for positions 13–25, and 79.17% (SD 9.32%) for positions 26–37. No significant differences between groups were observed (*p* = 0.87) (Figure 4B). All affiliation and position groups exceeded the 70% threshold for acceptable understandability.

### 3.5 Actionability

**Figure 5:**
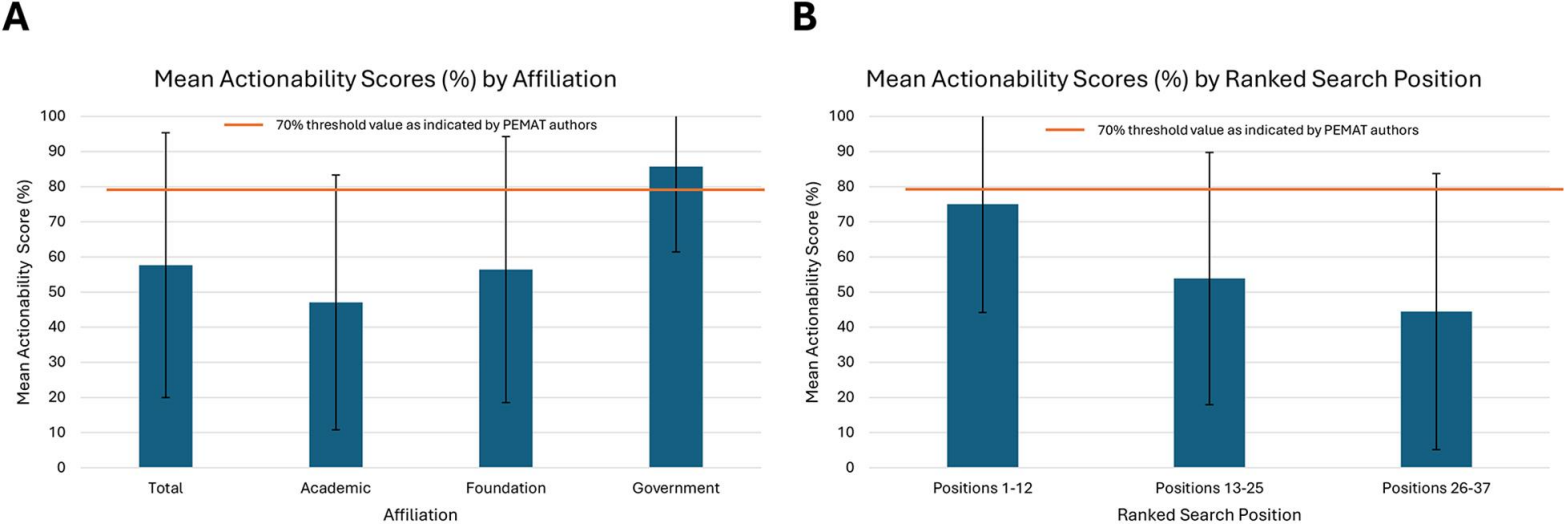
Mean PEMAT actionability scores with 70% recommendation threshold line indicated. **(A)** Mean total Actionability score and scores by affiliation (*p* = 0.07), **(B)** Mean Actionability scores by ranked search position (*p* = 0.13). PEMAT Actionability scores are expressed as a percentage of total criteria met (1–3).

Only 14 of the 37 websites in the study met the 70% threshold score for appropriate actionability. The mean actionability score was 57.66% (SD 37.69%) across all websites, far beneath the recommended threshold of 70%. Mean scores were 47.06% (SD 36.26%), 56.41% (SD 37.86%), and 85.71% (SD 24.28%) for academic, foundation, and government pages, respectively (Figure 5A). Although government sites were the only affiliation that demonstrated a mean actionability score above the 70% threshold, there was no statistically significant difference in actionability between academic, foundation, and government sites (*p* = 0.07). Mean actionability scores were 75% (SD 30.81%) for websites 1–12 by ranked search position, 53.85% (SD 35.90%) for the websites 13–25, and 44.44% (SD 39.28%) for the websites 26–37. Only the group of websites 1–12 by ranked search position met the 70% recommendation. The differences in mean actionability scores between ranked search position groups were not statistically significant (*p* = 0.13) (Figure 5B).

### 3.6 Readability

**Figure 6:**
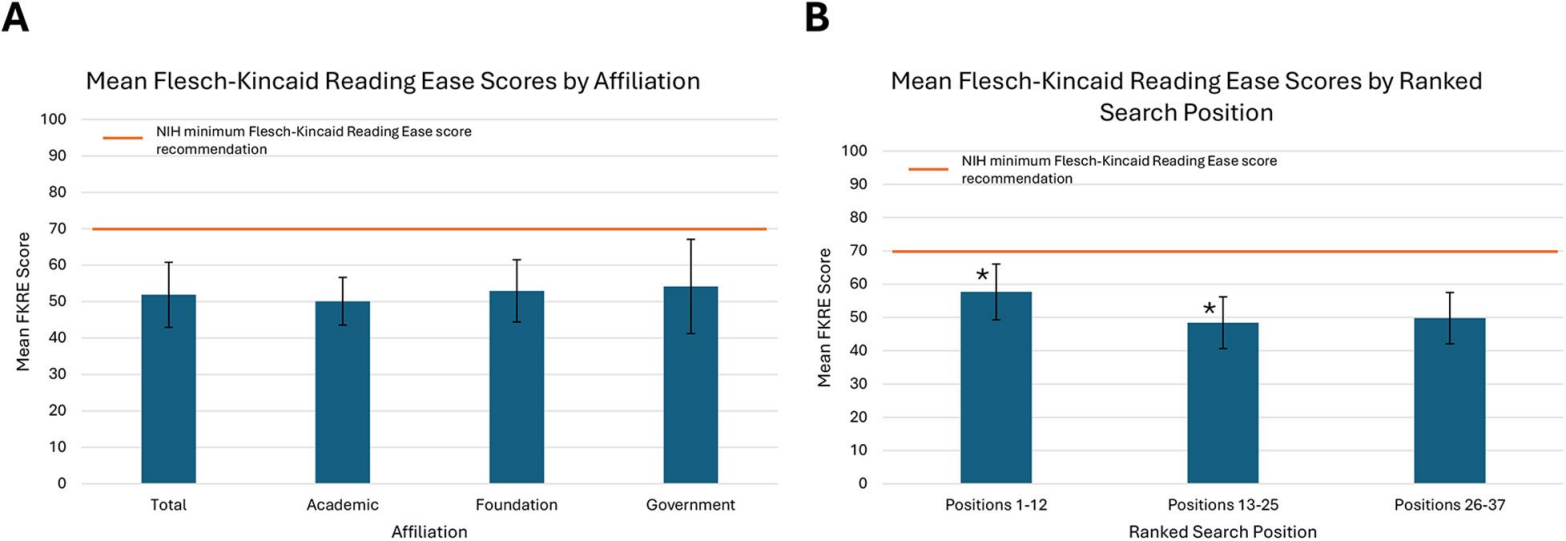
**(A)** Mean total Flesch-Kincaid Reading ease score and scores by affiliation with NIH recommended reading level indicated (*p* = 0.54), **(B)** mean Flesch-Kincaid Reading ease scores by ranked search position with NIH recommended reading level indicated (*p < 0.05).

19 of the 37 websites evaluated had a mean FKRE score equating to a college reading level or greater. The mean FKRE score across all web pages was 51.88 (SD 8.93), correlating to a 10th–12th grade reading level. The mean score for academic, foundation, and government pages was 50.11 (SD 6.55), 52.96 (SD 8.54), and 54.17 (SD 12.93), respectively, all of which equate to a 10th–12th grade reading level or above in the United States (Figure 6A). No significant differences were present between categories (*p* = 0.54). The top 12 web pages by search position had a mean FKRE score of 57.68 (SD 8.39), equating to a 10th–12th grade reading level. Websites ranked as search positions 13–25 had a mean FKRE score of 48.44 (SD 7.77), and websites ranked 26–37 had a mean FKRE score of 49.80 (SD 7.71), both of which equate to a college reading level. A significant difference was demonstrated between the 1–12 search positions group and the 13–25 search position group (*p* = 0.02), with the top 12 search positions having a higher mean FKRE score by nearly 10 points (Figure 6B).

### 3.7 Comprehensiveness

**Figure 7:**
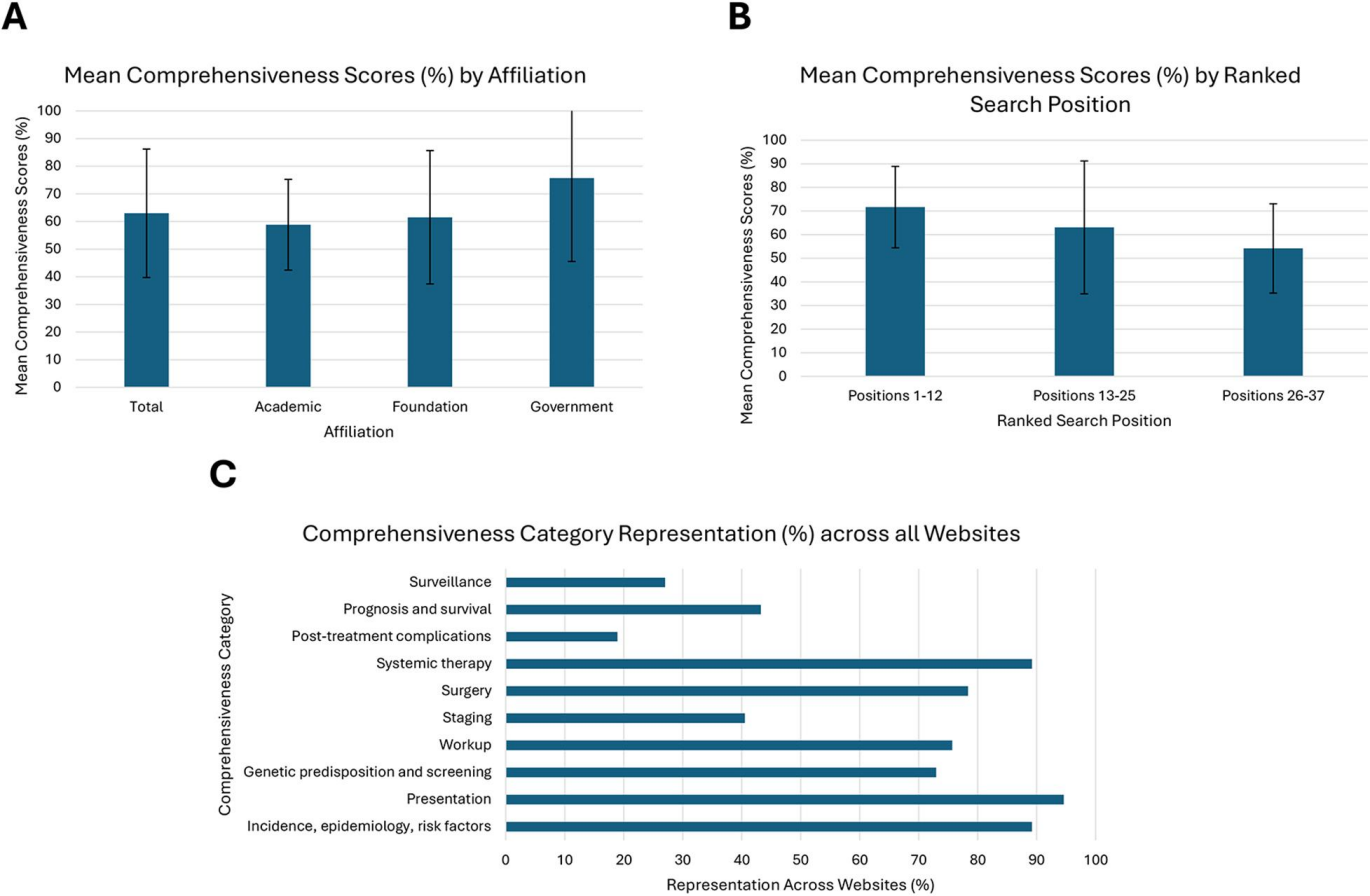
**(A)** Mean total comprehensiveness score and scores by affiliation (*p* = 0.28), **(B)** mean comprehensiveness scores by ranked search position (*p* = 0.19), **(C)** individual category representation across all websites (%). Comprehensiveness scores are expressed as a percentage of total criteria met (1–10).

Across all the assessed categories, the mean score for comprehensiveness was 62.98% (SD 23.23%). Academic, foundation, and government pages had a mean of 58.82% (SD 16.41%), 61.54% (SD 24.13%), and 75.71% (SD 30.17%), respectively (Figure 7A). There were no significant differences observed between groups (*p* = 0.28). When grouped by ranked average search position, positions 1–12 had a mean score of 71.67% (SD 17.24%), positions 13–25 had a mean of 63.08% (SD 28.12%), and positions 26–37 had a mean of 54.17% (SD 18.91%). No significant differences were present between position groups (*p* = 0.19) (Figure 7B). The three categories most represented across all web pages were “Incidence, epidemiology, risk factors”, which was present in 89.19% of evaluated websites, “Presentation”, present in 94.59% of websites, and “Systemic therapy”, present in 89.19% (Figure 7C). The two categories with the least representation were “Post-treatment complications”, present in 18.92% of evaluated websites, and “Surveillance”, present in 27.03% of websites (Figure 7C).

## 4 Discussion

The quality of the evaluated resources, as measured by the DISCERN scoring tool, was found to be adequate across different website affiliation categories and positions, with mean scores above 3. While most websites provided relevant information regarding the diagnosis, they frequently failed to address current knowledge limitations and the natural progression of the disease. Assessment of understandability using the PEMAT indicated that most websites met the recommended threshold of 70%, regardless of affiliation or position, suggesting acceptable clarity of presented information. However, the evaluation of actionability revealed significant deficits, with the overall mean actionability score falling below the 70% benchmark and only 14 of the 37 websites in the study meeting the 70% threshold score, indicating a lack of guidance on how patients might apply the information to their condition and empower them to advocate for their care. Readability analysis further demonstrated that the evaluated resources were written at a level far above that recommended for the public, with the mean readability at a 10th–12th reading level or above. Lastly, the analysis of comprehensiveness showed that most websites adequately addressed the epidemiology and symptomatology of gastric cancer. However, there was a notable lack of content concerning long-term management and post-treatment considerations. Overall, online resources for gastric adenocarcinoma generally provide relevant and understandable information, but they lack adequate patient actionability, appropriate readability, and comprehensive coverage of ongoing care management and disease progression.

Previous work investigating the online patient education resources for gastric cancer across various metrics has documented high readability levels, often around 10th grade or above, and only moderate completeness and accuracy (26, 27). However, gaps still exist in the evaluation of gastric cancer resources. For instance, a mixed-cancer resource evaluation utilizing a PEMAT and DISCERN comparison of English and Spanish materials did not isolate gastric cancer content or include key measures like readability and comprehensiveness (26). Likewise, although validated-tool studies have shown persistent problems with incomplete discussion of treatment risks and quality-of-life impacts or low quality scores, they were limited by sample size or lack of recency, and did not evaluate all domains such as understandability or actionability, underscoring the ongoing need for a larger, more holistic assessments specific to gastric cancer resources, as was performed in this study (28). Additionally, this more recent analysis indicates that many of the same deficiencies identified in the previous studies continue to persist in current websites.

This study also highlights that the shortcomings identified in the evaluation of gastric cancer resources mirror those identified in patient education materials for other cancer types. A study by Gulbrandsen et al. (36) demonstrated that, in evaluating 37 unique online patient resources for osteosarcoma, none of the resources met the threshold for appropriate reading level, as defined by the NIH, and only 1 site met the acceptable actionability threshold. Another study assessing web-based materials for acute myeloid lymphoma showed an adequate understandability score of 86%, but a mean actionability score of 63%, below the 70% threshold (37). These shortcomings in actionability across online patient education resources in several areas, including those identified in our study, demonstrate a general lack of practical guidance and patient-centered communication that supports individuals in taking informed, proactive roles in their healthcare. Patients seeking online sources to gain a better understanding of their health should be empowered to engage meaningfully in shared decision-making, whether through providing them with essential questions to ask their providers or through clear, actionable steps they can take to manage symptoms, adhere to treatment, or pursue further evaluation.

Using various readability formulas to evaluate the readability of online breast cancer education resources for patients, Gu et al. (38) found a mean readability score of almost double that of the recommended 6th-grade reading level as defined by the American Medical Association. Mean readability scores calculated for pancreatic cancer websites in a study by Storino et al. demonstrated higher-than-recommended reading levels for all website categories, especially for media-owned and university-based websites (23). However, this study also found that a higher reading level was associated with a higher accuracy (23). In a study investigating the readability of online patient education materials for chronic low back pain, mean FKRE and Gunning Fog scores indicated “very difficult” text requiring reading levels well above the recommended threshold (13). The findings in these works support the trend observed in the present study, indicating that the readability levels of online patient education resources consistently exceed the levels recommended for the general public. Given the substantial number of individuals who use the internet to seek information about their medical conditions, these resources must be written at a level that aligns with the average health literacy of the population. United States Census data from 2021 show that while 91.1% of U.S. adults aged 25 or older have earned a high school diploma, only 37.9% have attained a bachelor's degree or higher (39). Consequently, a significant portion of the population may struggle to fully comprehend online medical information written at a 10th–12th-grade level or above. While it is essential to improve the readability of online health resources, this effort must also be balanced with the need to preserve the accuracy and quality of the information presented.

In evaluating the comprehensiveness of information presented in online resources for breast, lung, prostate, and colorectal cancer, Li et al. (40) found that while risk factors, symptoms, and detection were adequately covered across sources, prognosis was often not addressed. In a similar study focusing on online information for cervical cancer, it was demonstrated that over 90% of resources reported etiology and risk factors, but less than 50% mentioned prognosis, side effects of treatment, and follow-up (41). These findings highlight similar information gaps as those noted in the present work, particularly regarding future health outlook and post-treatment management. Patients seeking comprehensive information should be provided with accessible and detailed resources that address these topics to enhance their understanding of the full continuum of cancer care.

One notable limitation of the study was the tools used to evaluate websites. While the DISCERN and PEMAT instruments provided structured guidelines and clearly defined criteria, these tools inherently involve a degree of subjectivity when assigning ratings to the various evaluation domains. The impact of this subjectivity was mitigated by having two independent reviewers assess each website and reach a consensus on the final scores; however, even with this approach, the evaluations may still have been influenced by individual reviewer bias and interpretive differences. Another limitation of this study is that readability was assessed using only the FKRE score, which relies primarily on sentence length and syllable count and may not fully capture textual complexity across different dimensions. In contrast, using multiple established tools, such as Gunning Fog, SMOG, ARI, Coleman–Liau, or Linsear Write and averaging their results could have provided a more robust and comprehensive assessment of readability, as these indices evaluate readability using distinct linguistic features and assumptions (42). Nonetheless, the Flesch–Kincaid Reading Ease was selected for this study as a widely validated and commonly applied measure of overall readability, and although incorporating additional indices might have yielded marginal variation in absolute scores, the overall comparative patterns and conclusions were unlikely to differ substantially given the strong correlations among these measures, as identified in a 2004 report by DuBay (43). Additionally, this study was limited to evaluating websites that contained written patient information. The inclusion of a broader range of patient education media—such as YouTube videos, infographics, or widely used social media platforms—could provide valuable insights into information sources that are more commonly accessed by specific patient populations and that may reveal additional criteria or communicative elements that contribute to either effective or ineffective patient education not fully encompassed in websites relying solely on written language. Drawing on bibliometric research in other fields, which has shown that resources with high social media attention may reach audiences beyond traditional academic metrics, incorporating analyses of online engagement could similarly illuminate the reach and impact of gastric cancer educational resources (44). Furthermore, all websites in this study were identified using the search term “stomach cancer”. While this term is arguably the most familiar and widely used among the public, it is possible that some patients might search using more clinical terminology, such as “gastric adenocarcinoma”, which could lead to different search results and alter the composition of websites included in the analysis. Lastly, the study only considered websites appearing within the top 50 search results for each browser and search engine combination. This limitation may have excluded potentially relevant or high-quality websites that appeared beyond the 50th position. Including a broader range of search results could have enhanced the comprehensiveness of the analysis and revealed additional gaps in the quality or accessibility of online patient education materials.

This study's strengths include its large-scale, contemporary evaluation of gastric cancer–specific online patient education resources using multiple validated tools to assess quality, understandability, actionability, readability, and comprehensiveness in a holistic manner. By isolating gastric cancer content and examining several domains not consistently evaluated in prior work, it fills a critical gap left by earlier, smaller, or mixed-cancer studies. Additionally, the use of standardized instruments with independent reviewers enhances methodological rigor and allows meaningful comparison with existing literature across cancer types.

Overall, findings from the present study highlight that, while online resources for gastric cancer generally offer relevant and understandable information, they fall short in key areas that are essential for effective patient education. These include limited actionability, poor readability, and incomplete coverage of topics such as long-term management and prognosis. Gastric cancer is a highly lethal disease, often diagnosed at an advanced stage with poor survival, requiring complex, multimodal treatment and long-term care (1–4). This combination creates unique information needs and decision-making challenges, especially given its variable symptoms, aggressive course, and the need for patients to understand nuanced treatment pathways and prognosis early in the disease course. To better support patients, future efforts should aim to prioritize the development of resources that are actionable, widely accessible, and comprehensive. Enhancing these aspects will empower individuals to engage more effectively in their care and shared decision-making.

In widening the scope of the present work, future research could explore emerging sources of online health information beyond traditional websites, including AI-driven tools and widely used video-based platforms such as YouTube. A recent survey found that while search engines are most used by patients, 21.2% reported using AI chatbots like ChatGPT and Microsoft Copilot (11). In contrast to the structured, text-based, and relatively stable nature of websites, as investigated in the present study, AI tools offer personalized conversational and rapidly updated responses, while platforms such as YouTube rely on audiovisual content. As AI use grows, it offers a more efficient way to access targeted information, and video-based platforms may enhance engagement and comprehension, but also pose a risk for misinformation. In one study, AI chatbots were used to analyze how patients might learn about low back pain by evaluating the readability, reliability, and quality of AI-generated answers to common patient questions, revealing that current AI explanations are often too complex and insufficiently reliable for effective patient education (45). Another study investigated the use of YouTube as an information source to evaluate how patients learn about percutaneous tracheostomy by analyzing the quality, reliability, and informational value of widely viewed videos, finding that most content was low quality and insufficient, with higher-quality information mainly provided by longer, clinician- or academically-produced videos (46). Together, these findings highlight interesting features regarding the quality and educational value of emerging digital health information platforms. Building on this work, studies assessing the quality, readability, and usefulness of AI-generated and video-based health information on gastric cancer could offer insights into its reliability for patient education. Additionally, evaluating how features of these differ from and could complement traditional websites may help create more accessible, patient-centered content that surpasses current standards.

## Data Availability

The raw data supporting the conclusions of this article will be made available by the authors, without undue reservation.
